# Successful implementation of a rater training program for medical students to evaluate simulated pediatric emergencies

**DOI:** 10.3205/zma001629

**Published:** 2023-06-15

**Authors:** Nadine Mand, Tina Stibane, Helmut Sitter, Rolf Felix Maier, Andreas Leonhardt

**Affiliations:** 1 Philipps University of Marburg, University Hospital Marburg, Department of Pediatrics, Marburg, Germany; 2 Philipps University of Marburg, Dr. Reinfried Pohl Center for Medical Education, Marburg, Germany

**Keywords:** simulation, medical students, pediatric life support

## Abstract

**Introduction:**

Simulation-based training is increasingly used in pediatrics to teach technical skills, teamwork, and team communication, and to improve potential deficiencies in pediatric emergency care. Team performance must be observed, analyzed, and evaluated by trained raters. The structured training of medical students for the assessment of simulated pediatric emergencies has not yet been investigated.

**Methods:**

We developed a rater training program for medical students to assess guideline adherence, teamwork, and team communication in simulated pediatric emergencies. Interrater reliability was measured at each training stage using Kendall tau coefficients.

**Results:**

In 10 out of 15 pairs of raters interrater reliability was moderate to high (tau>0.4), whereas it was low in the remaining 5 pairs of raters.

**Discussion:**

The interrater reliability showed good agreement between medical students and expert raters at the end of the rater training program. Medical students can be successfully involved in the assessment of guideline adherence as well as teamwork and team communication in simulated pediatric emergencies.

## Zusammenfassung

**Einleitung:**

Auf Simulation basierende Schulungsformate werden zunehmend in der Pädiatrie eingesetzt, um technische Fertigkeiten, Teamarbeit und Teamkommunikation zu trainieren und Defizite in pädiatrischen Notfallversorgungen zu verbessern. Geschulte Beobachter*innen sind notwendig, um dies im Rahmen simulierter Notfallsituationen zu erfassen, zu analysieren und zu beurteilen. Die strukturierte Schulung von Medizinstudierenden zur Beurteilung pädiatrischer Notfallsituationen im Rahmen von Simulationstrainings wurde bisher nicht untersucht.

**Methoden:**

Entwicklung eines mehrstufigen Beobachtertrainings für Medizinstudierende zur Bewertung der Leitlinienadhärenz, sowie der Teamarbeit und -kommunikation. Die Interrater-Reliabilität wurde in jeder Stufe des Trainings durch die Bestimmung von Kendall tau-Koeffizienten ermittelt.

**Ergebnisse:**

In 10 von 15 Beobachterpaaren wurde eine moderate bis hohe Interrater-Reliabilität gezeigt (tau>0,4), in den übrigen 5 Beobachterpaaren eine schwache Übereinstimmung.

**Diskussion:**

Die Interrater-Reliabilität zeigte nach Beendigung des Beobachtertrainings eine gute Übereinstimmung zwischen den Beobachter*innen. Medizinstudierende können demnach erfolgreich in die Evaluation der Leitlinienadhärenz sowie der Teamarbeit und -kommunikation einbezogen werden.

## 1. Introduction

Prehospital and intrahospital resuscitation in children occurs less frequently than in adults and is associated with high morbidity and mortality [1], [2], [3], [4]. For the majority of physicians working in pediatrics, routine in these stressful emergencies cannot be achieved. They often feel unconfident and unprepared during resuscitation [5], [6], [7], which has an impact on the delivery of emergency care [8], [9], [10]. 

Inadequate technical skills [8], [11], lack of teamwork, and communication errors are considered to be reasons for deficiencies in emergency care [12], [13]. Simulation-based training formats address these deficiencies in a targeted manner [14], [15]. However, in addition to structured development and embedding in a meaningful teaching concept [16], [17], effective simulation training requires accurate observation and evaluation of team performance [18]. This is essential for effective debriefing following a simulated or real emergency [19] and for scientific evaluation, quality assurance, and optimization of simulation-based training formats.

In addition to the selection of a valid and reliable assessment tool, specifically trained raters are essential to observe, analyze and evaluate technical skills, teamwork, and team communication in simulated emergencies [18], [20].

Physicians in the early stages of training or clinically inexperienced medical students were involved as raters with varying success [20], [21], [22], [23], [24], [25]. Previously published descriptions of rater training are inconsistent, and details on content are rarely provided [20], [21], [22], [23], [24]. Systematic training of medical students to assess simulated pediatric emergencies has not yet been investigated. This study describes the development, evaluation, and successful implementation of a rater training program for medical students to assess guideline adherence, teamwork, and team communication.

## 2. Methods

### 2.1. Assessment tools

We used the *performance evaluation checklist for pulseless ventricular tachycardia (PEC-PVT)* for evaluating guideline adherence (see attachment 1 ) [26]. The PEC-PVT evaluates the management of a simulated pediatric emergency with cardiovascular failure due to a shockable heart rhythm. It consists of 31 items, which are divided into three evaluation categories (task not performed; task performed partially, incorrectly, or with delay; and task performed completely). Each item is weighted between 1 and 5 according to its importance to treatment success. Thus the PEC-PVT illustrates the complexity of a resuscitation situation. The English version has been validated with German pediatric emergency teams [26]. It was translated into German by one of the original authors (EH), followed by a consensus on the final version between EH, the co-authors, and the principal investigator (NM). Based on the phases of the study scenario, individual items were specified, and structured by the principal investigator. To enable the most accurate evaluation of all items, a rater training handbook was developed, thus specifying the rating of each item (see attachment 2 ). 

Teamwork and team communication were evaluated using the *Teamwork Emergency Assessment Measure (TEAM)* [27]. Consisting of 11 items that evaluate aspects of team leadership, teamwork, and team communication on a 5-point Likert scale (“never/hardly ever” to “always/nearly always”), as well as a global assessment of teamwork (scale from 1 to 10), this checklist is easy to use. The validity and reliability of the TEAM are high [28], [29]. Since no German version had been published at the beginning of the rater training, the checklist was translated into German using forward-backward translation [30]. Based on the “TEAM Behavioural Markers” published by Cooper [31] and Rall's Crew Resource Management concept [32], a rater training handbook was designed to provide users with specific and observable “behavioral anchors” that represent the extreme ends of the TEAM observation scale [18]. 

### 2.2. Participants

Five medical students in intermediate to advanced training (7^th^ to 13^th^ semester) participated in the rater training. Two of the students had basic simulation knowledge as student tutors in medical education. One student already had experience in emergency medicine as a trained paramedic. All students were beginners in the structured observation and analysis of simulated emergencies.

Each student was trained in the use of one checklist. Training in the application of the specific Performance Evaluation Checklist (PEC-PVT) was conducted for two of the medical students, while three were trained in the application of the Team Emergency Assessment Measure (TEAM). 

### 2.3. Rater training

The rater training program was based on previously published framework structures for rater training [18], [33], [34] and included different formats such as self-study and interdisciplinary training. Three core training strategies being essential for the evaluation of observations were incorporated: *Rater Error Training* deals with typical observational errors and their avoidance, *Performance Dimension Training* teaches the recognition of desired behaviors for the item to be examined and in the *Frame of Reference Training*, variations in the quality of a desired behavior is demonstrated [33], [34]. 

Rater training took place over a period of six weeks and was carried out separately by the principal investigator for each of the two assessment tools.

#### Session 1: Conveying expertise

In external courses, the medical students were taught basic knowledge relevant to their assessment tool. Two students attended a two-day Pediatric Advanced Life Support (EPALS) course offered by the European Resuscitation Council, thus learning about the recognition of a critically ill child as well as life support in the event of respiratory and cardiovascular arrest. Three students took part in a one-day Crew Resource Management (CRM) course, learning aspects of team leadership, teamwork, and team communication.

#### Session 2: Self-study

The students familiarized themselves with the theoretical aspects of the above-mentioned core training strategies, and typical rater errors to achieve a general understanding of evaluating team performance. They also familiarized themselves with the assessment tools. 

#### Session 3: First video evaluation

There were two rater trainings: assessing team performance using the PEC-PVT was taught separately from using TEAM. After a structured summary of relevant rater errors followed by a group discussion, two video examples were individually rated by each medical student and the principal investigator. Examples of poor or acceptable team performance were chosen. The team performance was recorded using a paper-and-pencil version of the respective checklists. Subsequently, the individual items were discussed with special attention to those items with the lowest agreement between the medical students and the principal investigator, and reasons for substantial deviation were named. 

#### Session 4: Rater training handbook, second video evaluation

For the second video session, the above-mentioned two videos with poor and acceptable team performance, as well as three additional videos were evaluated independently using the rater training handbook. The ensuing discussion focused on the individual items with the least agreement. Here, too, training took place separately for the PEC-PVT and the TEAM.

#### Session 5: Pilot testing

Over a period of 4 weeks, the medical students and the principal investigator evaluated further nine videos using the checklists and the rater training handbook.

### 2.4. Statistics

In each phase of the rater training, interrater reliability between medical students and the principal investigator was examined pairwise by calculating the Kendall-tau-b correlation coefficient. The Kendall-tau coefficient measures the agreement of raters and thus quantifies the reliability of the observation system. This coefficient is a rank correlation coefficient and takes values between -1 and 1. This measure is suitable for ordinal data such as Likert scales. Only the ranking of the respective values is taken into account. Tau is determined using the individual items of the assessment tools [35]. A weak or low agreement is assumed with a Kendall-tau coefficient of ≤0.4, a moderate one with 0.41-0.7, and a high agreement with values >0.7 [36].

The calculated Kendall-tau correlation coefficients are based on two videos in rater training session 3, five videos in session 4, and nine videos in session 5. The number of videos was restricted due to the complexity of the rater training program. The pairwise matches were determined to evaluate the assessment of a student in comparison to the principal investigator. 

Differences in the assessment of individual items were investigated using Mann-Whitney-U tests (for the eleven individual items of TEAM and all items of the PEC-PVT) and t-tests (for the global evaluation of TEAM). Data analysis was carried out using SPSS V24 (Armonk, NY: IBM Corp.).

### 2.5. Ethics

The rater training program was developed as part of the study: “Quality assurance of pediatric emergency care through in-house simulation training at Hessian Children’s Hospitals”. The Ethics Committee of the Philipps University Marburg approved this study (ref. no. 172/16). 

## 3. Results

All five medical students completed the rater training program. Sixteen videos (two videos from rater training session 3, five videos from session 4, and nine videos from session 5) of five pairs of raters each (one student and the principal investigator) were evaluated. With five pairs of observations and three video sessions, 15 Kendall-tau coefficients could be calculated (see table 1 (Tab. 1)). 

Five of the 15 Kendall-tau coefficients showed weak agreement between medical students and the principal investigator, five moderate, and five high agreement (see table 1 (Tab. 1)). In three cases, the agreement between the first and second video assessments improved (observation pairs 1, 3, and 5).

The U- and t-tests performed did not show any significant differences between medical students and the principal investigator, i.e. no individual items could be identified in which the evaluation differed between beginners and expert.

## 4. Discussion

We present a rater training program enabling medical students to evaluate simulated pediatric emergencies. Different training formats and core training strategies were combined [33], [34]. We aimed for evaluating and implementing a rater training program for consecutive studies based on video evaluations of team performance for both experts and trained beginners.

Being usually beginners in assessing team performance, medical students are rarely chosen for evaluating complex simulated emergencies. Freytag and colleagues involved students in the evaluation of teamwork and team communication of simulated emergencies in medical training after a rater training [20]. Interrater reliability was moderate, due to a more lenient assessment by the students as compared to the experienced co-raters [20], [37]. Initial interrater reliability before participation in rater training is not provided. Evans et al. investigated the use of a checklist for the assessment of invasive procedures by students after four hours of rater training and found a good agreement between the ratings of beginners and experts [22]. They concluded that teaching observational skills is possible even without prior background knowledge of the raters [22]. 

We demonstrated that medical students as beginners in structured behavioral observation can achieve good interrater reliability in the context of a rater training program. This was evident both in the evaluation of complex behaviors such as teamwork and team communication and in the assessment of technical skills such as guideline adherence. After a structured and detailed training of both evaluation strategies and theoretical and practical aspects of pediatric life support (rater training sessions 1 to 3), a good agreement in the evaluation of guideline adherence between beginners and experts was demonstrated, even before the actual video evaluation. Subsequently, the use of rater training handbooks improved the interrater reliability of those evaluating teamwork and team communication (session 4). This *frame of reference* training has been shown in previous studies to be particularly effective in rater training [34]. Extended rater training beyond the evaluation of a few exercise videos (session 5) did not further improve interrater reliability. 

Medical students can therefore support experts in the observation, analysis, and evaluation of simulated emergencies. This is relevant in the context of limited human resources, as simulation-based training formats are increasingly used in medical education and training. In addition to the training of technical and non-technical skills [38], simulation training can address relevant aspects of patient safety [39], [40] and identifies recurring and often avoidable treatment errors in the management of emergencies [41], [42], [43]. This also requires adequate training of the experts, i.e. teaching physicians. In addition to medical expertise, they need to be trained in core evaluation strategies, as well as in typical rater errors.

This study has several limitations. 

The Team *Emergency Assessment Measure (TEAM)* was originally developed for experts, not for use by beginners in behavioral observation [27], [44]. Since no pairwise video evaluation was carried out before the start of the rater training program, the construct validity of this checklist is not known for beginners. However, an effect of the rater training can be assumed because 2 out of 3 rater pairs evaluating the TEAM showed improvements in interrater reliability.

The evaluation of the specific performance evaluation checklist (PEC-PVT) showed better interrater reliability in this study as compared to the original publication [26]. While translating the original checklist into German, we detected several ambiguously phrased items. These items were clarified in the German version, which could have improved the interrater reliability. 

The rater training presented here is very personnel- and time-consuming and therefore not easily transferable to other studies. Since we could not demonstrate any improvement in interrater reliability in session 5 of the rater training program, training beyond a few exercise videos does not seem to be of any advantage. 

## 5. Conclusion

In summary, we were able to demonstrate that medical students can evaluate technical skills, as well as teamwork and team communication in complex simulated emergencies after completing a rater training program. The rater training should be structured and contain established core training strategies. Extended training beyond the use of a few exercise videos does not seem necessary.

## Funding

The Hessian Ministry of Social Affairs and Integration financed pediatric emergency training at Hessian children’s hospitals, in the context of which the study videos were made.

Open Access funding is provided by the Open Access Publishing Fund of Philipps-University Marburg with the support of the Deutsche Forschungsgemeinschaft (DFG, German Research Foundation).

## Competing interests

The authors declare that they have no competing interests. 

## Supplementary Material

PEC-PVT rater training handbook

PEC-PVT

## Figures and Tables

**Table 1:**
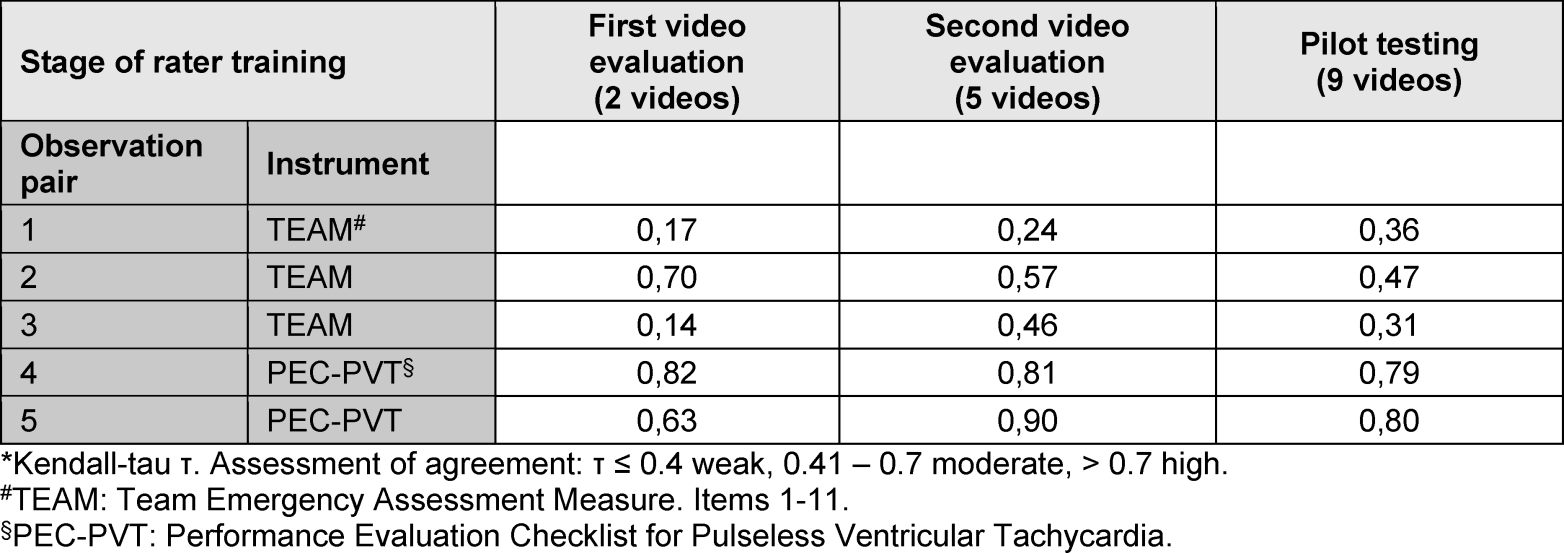
tau* for the agreement between students and the principal investigator
