# Supplementary material for: Successful implementation of a rater training program for medical students to evaluate simulated pediatric emergencies
Source: GMS J Med Educ. 2023 Jun 15;40(4):Doc47. doi: 10.3205/zma001629 (PMC10407587; doi:10.3205/zma001629)
Supplement: PEC-PVT rater training handbook [file JME-40-47-s-001.pdf]

Attachment 1: PEC-PVT rater training handbook

Introduction

The presented Performance Evaluation Checklist (PEC) is based on the PEC-PVT published by Schmutz et al. [26]. It was translated into German by one of the original authors (Ellen Heimberg = EH), followed by consensus on the final version between EH, the co-authors, and the principal investigator (Nadine Mand = NM).

The finalized PEC has been structured according to the study scenario phases below. For each item 0, 1, or 2 points can be awarded: 0 points if a task is not performed at any time, 1 point if a task is performed partially, incorrectly, or with delay, 2 points if a task is performed completely. The rater training handbook is intended to specify when points can be awarded and thus contributes to a performance evaluation that is as precise as possible.

Study scenario

General information

The initial team of one nurse and one doctor receives a short, precise, and standardized patient case history from the instructor (depending on the scenario, the instructor portrays a parent, an emergency physician, or a nurse). After that, scenario time is started. Further questions from the team about the patient's medical history or vital signs can be asked during the scenario. Up to three additional team members can be called in for help.

Scenario

The initial state of the patient is a hemodynamically compensated shock. After a total of 2 minutes, the patient is apneic and pulseless due to a shockable heart rhythm (ventricular tachycardia or ventricular fibrillation). Regardless of the teams´ interventions, the patient has a *"return of spontaneous circulation"* (ROSC) after another 8 minutes. The team has another 2 minutes to plan additional actions. The scenario thus lasts a total of 12 minutes.

Learning objectives

Phase 1: Patient in hemodynamically compensated shock (duration 2 min)

- ABC evaluation, recognizing the critically ill patient with the diagnosis of "shock"
- Asking for help
- Anticipating clinical deterioration

Phase 2: Patient in apnea with pulseless, shockable heart rhythm (duration 8 min)

- Guideline adherent resuscitation (ventilation / CPR)
- Defibrillation (at least 3 shocks), drugs (epinephrine and amiodarone)
- Note: ROSC occurs after 8 minutes regardless of the tasks performed. Phase 2 can be shortened if the team has defibrillated 3 times, and administered epinephrine and amiodarone at least once.

Phase 3: Patient in ROSC (duration 2 min)

- Planning of further actions: i.e. considering advanced airway, contacting the pediatric intensive care unit
- Identification and treatment of reversible causes

1. General tasks and patient evaluation

All tasks should be performed within the first 2 minutes after contact with the patient, otherwise, they are considered "performed with delay". *For exceptions, see specific items.*

1.1. Put on protective gloves

1 point	2 points
• Not all team members put on protective gloves<br>OR<br>• Team members put on protective gloves after the first initial 2 minutes or after tending to the patient	• All team members who are involved up to this point put on protective gloves within 2 minutes and before tending to the patient<br>AND<br>• Team members who arrive later than 2 minutes after the scenario has started put on protective gloves before tending to the patient

1.2. Equipment check

1 point	2 points
• Checking equipment for bag-mask ventilation is incomplete<br>OR<br>• Checking is done after using it on the patient	• Checking equipment for bag-mask ventilation is complete (oxygen connected to flowmeter and bag, flow "turned up"; bag size, mask size, and seal are correct)<br>AND<br>• Checking the above-mentioned equipment before using it on the patient

1.3. Connect monitors

1 point	2 points
• Only 1 or 2 out of 3 (SpO_2, ECG, blood pressure) are established<br>OR<br>• Established after the initial 2 minutes	• All 3 (SpO_2, ECG, blood pressure) are correctly established<br>AND<br>• Established within 2 minutes

1.4. Call for help

1 point	2 points
• Senior physicians, more experienced colleagues, or additional assistants (doctors or nurses) are called later than **3 minutes** after initial contact with the patient<br>OR<br>• Senior physicians, more experienced colleagues, or additional assistants (doctors or nurses) are only called in on the advice of the instructors	• Senior physicians, more experienced colleagues, or additional assistants (doctors or nurses) are called within **3 minutes** of initial contact with the patient

1.5. ABC evaluation

1 point	2 points
• Not all of the following aspects were checked:<br>A/B: respiratory rate, SpO_2, tidal volume<br>C: capillary refill time (CRT), heart rate, blood pressure<br>D: Consciousness<br>OR<br>• Complete evaluation after the first 2 minutes	• All of the following aspects were checked:<br>A/B: respiratory rate, SpO_2, tidal volume<br>C: CRT, heart rate, blood pressure<br>D: Consciousness<br>AND<br>• Complete evaluation within 2 minutes

1.6. Oxygen	
**1 point**	**2 points**
• Application of less than 100% oxygen, e.g. via nasal cannula or blow-by oxygen<br><br>If 100% O_2 is applied after the onset of apnea, "0" points are awarded.	• Application of 100% oxygen, e.g. via a venti mask or by means of mask bag ventilation supporting breathing<br>AND<br>• Before the onset of apnea

1.7. Inform team members about diagnosis	
**1 point**	**2 points**
• Non-specific: e.g. "critically ill patient", "volume depletion" or stating symptoms including assessment (e.g. "the patient has a prolonged CRT, I don't like it") *<br>OR<br>• Communication with individual team members, not the whole team is informed<br>OR<br>• Later than 2 min after initial contact with the patient<br><br>*in the case of a list of symptoms without assessment, "0" points are awarded	• Specific: "The patient is in (compensated) shock"<br>AND<br>• Communication with the entire team currently present<br>AND<br>• Within 2 min after initial contact with the patient

2. Evaluation and treatment AFTER the onset of apnea and cardiac arrhythmia

2.1. Open / free / assess the airway	
**1 point**	**2 points**
• Performed incompletely or later than 30sec after apnea occurs<br>OR<br>• "Apnea" is not communicated to the team	• Completely and within 30sec after apnea occurs<br>AND<br>• "Apnea" is communicated to the team

2.2. Start bag-mask ventilation (BMV)	
**1 point**	**2 points**
• Later than 30sec after apnea occurs	• Within 30sec after apnea occurs

2.3. Optimize ventilation	
**1 point**	**2 points**
• Later than 30sec after starting ventilation<br>OR<br>• No verbalization about the effectiveness of bag-mask ventilation (auscultation)<br>OR<br>• No optimization of BMV within 30sec if necessary	• Within 30 seconds after starting ventilation<br>AND<br>• Verbalization about the effectiveness<br>AND<br>• Optimization of BMV within 30sec, if necessary (reposition of the head, 2-person ventilation, airway adjuncts)

2.4. Check pulse OR check for signs of life	
**1 point**	**2 points**
• Checking for a pulse OR checking for signs of life* occurs later than 30sec after the onset of cardiac arrhythmia<br>OR<br>• Pulselessness / Lifelessness is not communicated to the team<br><br>Based on the SpO_2 curve results in "0" points<br><br>*signs of life: spontaneous movement, coughing, or normal breathing	• Within 30 seconds after the onset of cardiac arrhythmia (checking for a pulse must be performed on the carotid artery, brachial artery, radial artery, or femoral artery OR asystole is auscultated OR absence of signs of life is verbalized)<br>AND<br>• Pulselessness / Lifelessness is communicated to the team

2.5. Identify ECG rhythm	
**1 point**	**2 points**
• ECG rhythm is checked BUT not verbalized<br>OR<br>• ECG rhythm is checked later than 30sec after the onset of cardiac arrhythmia OR wrong heart rhythm is verbalized	• Within 30sec after the onset of cardiac arrhythmia<br>AND<br>• Correct ECG rhythm is verbalized
**2.6. Start cardiopulmonary resuscitation (CPR)**	
**1 point**	**2 points**
• Later than 30sec after the onset of cardiac arrhythmia<br>OR<br>• Poor CPR technique: pressure depth and frequency that deviate from guidelines, inadequate chest relief, incorrect pressure point/rhythm, inadequate technique (e.g. arms not extended, not positioned vertically above patients)	• Within 30sec after the onset of cardiac arrhythmia<br>AND<br>• Good CPR technique: adequate pressure depth and frequency, adequate chest relief, correct pressure point/rhythm, adequate technique (e.g. arms outstretched, positioned vertically above patients)
**2.7. Prepare defibrillation**	
**1 point**	**2 points**
• Initiated later than 30 sec after the onset of cardiac arrhythmia, i.e. preparation started	• Initiated within 30 sec after the onset of cardiac arrhythmia, i.e. preparation started
**2.8. IV/IO infusion**	
**1 point**	**2 points**
• Completed later than 120sec after the onset of cardiac arrhythmia<br>OR<br>• More than 2 intravenous attempts taking more than 60sec	• Completed within 120sec after the onset of cardiac arrhythmia<br>AND<br>• Successful within 60sec with a maximum of two intravenous attempts OR intraosseous vascular access established
**2.9. 1st Defibrillation**	
**1 point**	**2 points**
• Is performed later than 90sec after the onset of cardiac arrhythmia<br>OR<br>• Incorrect dose (less than 4 J/kg or rounded down) in relation to the actual or the estimated patient's body weight (e.g. weight = 2 x (age+4))<br>OR<br>• Incorrect mode (i.e. attempting synchronization)<br>OR<br>• Not carried out correctly (position of the paddles not correct, procedure not correct)	• Is performed within 90sec after the onset of cardiac arrhythmia<br>AND<br>• Correct dose (4 J/kg or rounded up) in relation to the actual or the estimated patient's body weight (e.g. weight = 2 x (age+4))<br>AND<br>• Correct mode (i.e. no synchronization)<br>AND<br>• Carried out correctly (position of the paddles correct, procedure correct: loading, warning, checking safety distance, defibrillating)
**2.10. Continue CPR**	
**1 point**	**2 points**
• Started later than 10sec after defibrillation<br>OR<br>• Incorrect CPR technique (see point 2.6.)<br>OR<br>• The person performing CPR has not been changed<br>OR<br>• Prior pulse control has been performed	• Started within 10sec of defibrillation<br>AND<br>• Correct CPR technique (see point 2.6.)<br>AND<br>• The person performing CPR has been changed<br>AND<br>• Without prior pulse control

**2.11. Reassess cardiac rhythm**	
**1 point**	**2 points**
• Cardiac rhythm was reassessed, but not verbalized<br>OR<br>• The wrong cardiac rhythm was verbalized (non-shockable instead of shockable)<br>OR<br>• Reassessing and verbalizing was performed less than 100sec or more than 120sec after the 1st shock	• The correct cardiac rhythm was verbalized<br>AND<br>• Reassessing and verbalizing were performed between 100 and 120sec after the 1st shock OR after 10 correct CPR cycles
**2.12. 2nd defibrillation ***	
**1 point**	**2 points**
• Is performed less than 100sec or more than 120sec after 1st shock<br>OR<br>• Incorrect dose in relation to the actual or the estimated patient's body weight (see point 2.9.)<br>OR<br>• Incorrect mode (i.e. attempting synchronization)<br>OR<br>• Not carried out correctly (position of the paddles not correct, procedure not correct)	• Is performed between 100 and 120sec after 1st shock OR after 10 correct CPR cycles<br>AND<br>• Correct dose (4 J/kg or rounded up) in relation to the actual or the estimated patient's body weight (see point 2.9.)<br>AND<br>• Correct mode (i.e. no synchronization)<br>AND<br>• Carried out correctly (position of the paddles correct, procedure correct: loading, warning, checking safety distance, defibrillating)
**2.13. Continue CPR**	
**1 point**	**2 points**
• Analogous to point 2.10.	• Analogous to point 2.10.
**2.14. Reassess cardiac rhythm**	
**1 point**	**2 points**
• Analogous to point 2.11.	• Analogous to point 2.11.
**2.15. 3rd defibrillation ***	
**1 point**	**2 points**
• Is performed less than 100sec or more than 120sec after 2nd shock<br>OR<br>• Incorrect dose in relation to the actual or the estimated patient's body weight (see point 2.9.)<br>OR<br>• Incorrect mode (i.e. attempting synchronization)<br>OR<br>• Not carried out correctly (position of the paddles not correct, procedure not correct)	• Is performed between 100 and 120sec after 2nd shock OR after 10 correct CPR cycles<br>AND<br>• Correct dose (4 J/kg or rounded up) in relation to the actual or the estimated patient's body weight (see point 2.9.)<br>AND<br>• Correct mode (i.e. no synchronization)<br>AND<br>• Carried out correctly (position of the paddles correct, procedure correct: loading, warning, checking safety distance, defibrillating)
***Note:** If sinus rhythm occurs before the 2nd or 3rd shock (as scenario phase 2 ends due to scenario algorithm), continue at point 2.17.! If more than 3 shocks are performed, intermediate rhythm reassessments/CPR are skipped in evaluation: continue at point 2.17.!	
**2.16. Continue CPR**	
**1 point**	**2 points**
• Analogous to point 2.10.	• Analogous to point 2.10.

2.17. Reassess cardiac rhythm (after the third OR last shock)	
**1 point**	**2 points**
• Cardiac rhythm is reassessed, but not verbalized<br>OR<br>• The wrong cardiac rhythm is verbalized (not "sinus rhythm")<br>OR<br>• Reassessing and verbalizing are performed after less than 100sec or more than 120sec after the 3rd or last (in case of more than 3) shock	• The correct cardiac rhythm (sinus rhythm) was verbalized<br>AND<br>• Reassessing and verbalizing were performed between 100 and 120sec after the 3rd or last (in case of more than 3) shock OR after 10 correct CPR cycles

2.18. Check pulse OR check for signs of life	
**1 point**	**2 points**
• Checking for a pulse OR checking for signs of life* is performed less than 100sec or more than 120sec after the 3rd or last shock<br>OR<br>• Checking for a pulse OR checking for signs of life is not verbalized<br><br>Based on the SpO_2 curve results in "0" points<br><br>*signs of life: spontaneous movement, coughing, or normal breathing	• Checking for a pulse (carotid artery, brachial artery, radial artery, or femoral artery) OR checking for signs of life is performed between 100 and 120sec after the 3rd or last (in case of more than 3) shock OR after 10 correct CPR cycles<br>AND<br>• Pulselessness / Lifelessness is communicated to the team

2.19. Epinephrine	
**1 point**	**2 points**
• Incorrect dose administered in relation to the actual or estimated body weight<br>OR<br>• Administered before 3rd shock	• Correct dose (10µg/kg) administered in relation to the actual or estimated body weight via a vascular access<br>AND<br>• Administered after the 3rd shock

2.20. Amiodarone	
**1 point**	**2 points**
• Incorrect dose administered in relation to the actual or estimated body weight<br>OR<br>• Administered before 3rd shock	• Correct dose (5mg/kg) administered in relation to the actual or estimated body weight via a vascular access<br>AND<br>• Administered after the 3rd shock

2.21. Measure blood pressure or Capillary Refill Time (CRT)	
**1 point**	**2 points**
	• After ROSC

3. Identification and treatment of reversible causes

3.1. H & T evaluation	
**1 point**	**2 points**
• Incompletely done*	• Completely done*<br>AND<br>• Treatment performed as necessary<br><br>***„H":** Hypoxia, Hypovolemia, Hypo-/Hyperkalaemia / metabolic disorder, Hypothermia<br>**"T":** Toxic agents, Tension pneumothorax, Tamponade (cardia), Thrombosis (coronary or pulmonary)

3.2. Blood sampling (blood gases, blood sugar, electrolytes)	
**1 point**	**2 points**
• Done, but not verbalized<br>OR<br>• Incompletely done	• Performed AND verbalized AND complete

3.3. Planning further actions	
**1 point**	**2 points**
	• Done (e.g. considering advanced airway, contacting the pediatric intensive care unit, cardiologist)

If no shock is performed: Point 2.9. to 2.16. = 0 points each
