# Supplementary material for: Successful implementation of a rater training program for medical students to evaluate simulated pediatric emergencies
Source: GMS J Med Educ. 2023 Jun 15;40(4):Doc47. doi: 10.3205/zma001629 (PMC10407587; doi:10.3205/zma001629)
Supplement: PEC-PVT [file JME-40-47-s-002.pdf]

Attachment 2: PEC-PVT

Attachment 2 to Mand N, Stibane T, Sitter H, Maier RF, Leonhardt A. *Successful implementation of a rater training program for medical students to evaluate simulated pediatric emergencies*. GMS J Med Educ. 2023;40(4):Doc47. DOI: 10.3025/zma001629

PEC-PVT

Datum: **Video-Nr:** **Untersucher:**

1	**Allgemeine Maßnahmen und Evaluation**	**0 P.**		**1 Punkt**	**2 Punkte**	**Time**	**W**
1-1	Handschuhe anziehen	□	ø	□ nicht alle ODER nicht rechtzeitig	□ alle Beteiligten UND rechtzeitig		2
1-2	Ausrüstung überprüfen	□	ø	□ unvollständig ODER nicht rechtzeitig	□ vollständig UND rechtzeitig	**3 min**	4
1-3	Monitore anschliessen	□	ø	□ unvollständig ODER nicht rechtzeitig	□ vollständig UND rechtzeitig		4.5
1-4	Hilfe rufen	□	ø	□ nicht rechtzeitig ODER erst nach Empfehlung des Schauspielers	□ rechtzeitig		4.5
1-5	ABC-Evaluation	□	ø	□ unvollständig ODER nicht rechtzeitig	□ vollständig UND rechtzeitig		5
1-6	Sauerstoff	□	ø	□ nasale Kanüle (nicht 100%)	□ 100% Sauerstoff verabreicht		5
1-7	Mitteilung der Diagnose an den Rest des Teams	□	ø	□ unspezifisch ODER nicht rechtzeitig	□ spezifisch UND rechtzeitig		5

2	**Evaluation und Behandlung NACH Einsetzen Apnoe/Rhythmusstörung**	**0 P.**		**1 Punkt**	**2 Punkte**	**Time**	**W**
2-1	Atemwege öffnen/inspizieren/bewerten	□	ø	□ > 30 sec ODER keine Verbalisierung	□ < 30 Sek. UND verbalisiert		5
2-2	Beginn Beutel-Masken-Beatmung	□	ø	□ > 30 Sek.	□ < 30 Sek.		5
2-3	Überprüfen Effektivität Beutel-Masken-Beatmung	□	ø	□ > 30 sek ODER keine Verbalisierung	□ < 30 sek nach Beginn Beatmung UND Verbalisierung UND Optimierung, falls notwendig		5
2-4	Pulskontrolle	□	ø	□ > 30 Sek. ODER nicht verbalisiert	□ < 30 Sek. UND verbalisiert		5
2-5	EKG Rhythmus identifizieren	□	ø	□ Rhythmus gecheckt ABER nicht verbalisiert	□ korrekten Rhythmus verbalisiert		4.5
2-6	Start CPR	□	ø	□ > 30 Sek. nach Eintreten Pulslosigkeit ODER schlechte CPR Technik	□ < 30 Sek. nach Eintreten Pulslosigkeit UND gute CPR Technik		5
2-7	Defibrillator vorbereiten	□	ø	□ > 30 Sek. nach Eintreten Pulslosigkeit	□ < 30 Sek. nach Eintreten Pulslosigkeit		5
2-8	IV/IO Infusion etablieren	□	ø	□ > 120 Sek. nach Eintreten Pulslosigkeit	□ < 120 Sek. nach Eintreten Pulslosigkeit		4.5
2-9	Defibrillation (erste)	□	ø	□ > 90 Sek. nach Eintreten Pulslosigkeit ODER inkorrekte Dosis ODER inkorrekter Modus / Ablauf	□ < 90 Sek. nach Eintreten Pulslosigkeit UND korrekte Dosis UND korrekter Modus / Ablauf		5
2-10	CPR (fortführen)	□	ø	□ > 10 Sek. nach 1. Defibrillation ODER schlechte CPR Technik	□ < 10 Sek. nach 1. Defibrillation UND gute CPR Technik		5
2-11	Rhythmuskontrolle	□	ø	□ Rhythmus gecheckt, aber nicht verbalisiert ODER < 100 Sek. bzw. > 120 Sek nach 1. Defibrillation	□ korrekten Rhythmus verbalisiert UND zwischen 100 und 120 Sek. nach 1. Defibrillation		5
2-12	Defibrillation (zweite)	□	ø	□ < 100 Sek. ODER > 120 Sek. nach 1. Defibrillation ODER inkorrekte Dosis ODER inkorrekter Modus / Ablauf	□ zwischen 100 und 120 Sek. nach 1. Defibrillation ODER nach 10 CPR Zyklen UND korrekte Dosis UND korrekter Modus / Ablauf		5
2-13	CPR (fortführen)	□	ø	□ > 10 Sek. nach 2. Defibrillation ODER schlechte CPR Technik	□ < 10 Sek. nach 2. Defibrillation UND gute CPR Technik		5
2-14	Rhythmuskontrolle	□	ø	□ Rhythmus gecheckt, aber nicht verbalisiert ODER < 100 Sek. bzw. > 120 Sek nach 2. Defibrillation	□ korrekten Rhythmus verbalisiert UND zwischen 100 und 120 Sek. nach 2. Defibrillation		5
2-15	Defibrillation (dritte)	□	ø	□ < 100 Sek. ODER > 120 Sek. nach 2. Defibrillation ODER inkorrekte Dosis ODER inkorrekter Modus /Ablauf	□ zwischen 100 und 120 Sek. nach 2. Defibrillation ODER nach 10 CPR Zyklen UND korrekte Dosis UND korrekter Modus / Ablauf		5
2-16	CPR (fortführen)	□	ø	□ > 10 Sek. nach der 3. Defibrillation ODER schlechte CPR Technik	□ < 10 Sek. nach 3. Defibrillation UND gute CPR Technik		5
2-17	Rhythmuskontrolle	□	ø	□ Rhythmus gecheckt, aber nicht verbalisiert ODER <100 Sek. bzw. >120 Sek. nach 3. (bzw. letzter) Defibrillation	□ korrekten Rhythmus (SR) verbalisiert UND zwischen 100 und 120 Sek. nach 3. (bzw. letzter) Defibrillation		5
2-18	Pulskontrolle	□	ø	□ Puls gecheckt, aber nicht verbalisiert ODER <100 Sek. bzw. >120 Sek. nach 3. (bzw. letzter) Defibrillation	□ Puls gecheckt UND verbalisiert UND zwischen 100 und 120 Sek. nach 3. (bzw. letzter) Defibrillation		5
2-19	Adrenalin	□	ø	□ Falsche Dosis ODER vor der 3. Defibrillation verabreicht	□ IV / IO korrekte Dosis verabreicht nach 3. Defibrillation		5
2-20	Amiodaron	□	ø	□ Falsche Dosis ODER vor der 3. Defibrillation verabreicht	□ IV / IO korrekte Dosis verabreicht nach 3. Defibrillation		4
2-21	Blutdruck ODER Recap Zeit messen	□	ø	□	□ nach ROSC		3

3	**Suche nach reversiblen Ursachen**	**0 P.**		**1 Punkt**	**2 Punkte**	**W**
3-1	4Hs & HITS-Evaluation	□	ø	□ Unvollständig erledigt	□ Komplett durchgeführt und falls nötig Gegenmassnahmen eingeleitet	4.5
3-2	Blutentnahme für BGA, Glukose, Elektrolyte	□	ø	□ Erledigt, aber nicht verbalisiert	□ durchgeführt UND verbalisiert	3.5
3-3	Frühzeitig weitere Behandlung anbahnen	□	ø	□	□ erledigt	3

**Zeit bis 1. Defibrillation:** sec	**Zeit bis CPR:** sec	**Zeit bis MB-Beatmung:** sec	**Gewicht:** kg
**Dosis 1./2./3. Def.:** J	**Dosis Adrenalin:** µg	**Dosis Amiodaron:** mg	
